# Association of Glycemic Status With Impaired Lung Function Among Recipients of a Health Screening Program: A Cross-Sectional Study in Japanese Adults

**DOI:** 10.2188/jea.JE20140016

**Published:** 2014-09-05

**Authors:** Yusuke Kabeya, Kiyoe Kato, Masuomi Tomita, Takeshi Katsuki, Yoichi Oikawa, Akira Shimada

**Affiliations:** Department of Internal Medicine, Saiseikai Central Hospital, Tokyo, Japan; 東京都済生会中央病院

**Keywords:** diabetes, glycemic status, lung function

## Abstract

**Background:**

The dose-response relationship between glycemic status and lung function has not been thoroughly investigated. We hypothesized that there are continuous and inverse associations between glycemic measures and lung function tests and examined the hypothesis in Japanese adults.

**Methods:**

We cross-sectionally investigated associations of hemoglobin A1c (HbA1c) and fasting plasma glucose (FPG) with forced vital capacity (FVC) and forced expiratory volume in one second (FEV1) in 3161 adults who participated in a health screening from 2008 to 2011. The study participants included both diabetic and non-diabetic adults. Multiple linear regression analyses were performed to examine the associations.

**Results:**

Inverse associations were observed in both sexes, which were attenuated in women after adjustment for multiple variables. A 1% absolute increase in HbA1c was associated with a −52-mL (95% confidence interval [CI] −111 to 8 mL) difference in FVC and a −25-mL (95% CI −75 to 25 mL) difference in FEV1 in women, and a −128-mL (95% CI −163 to −94 mL) difference in FVC and a −73-mL (95% CI −101 to −44 mL) difference in FEV1 in men. A 10-mg/dL increase in FPG was associated with a −11-mL (95% CI −29 to 8 mL) difference in FVC and a −8-mL (95% CI −24 to 7 mL) difference in FEV1 in women, and a −32-mL (95% CI −44 to −21 mL) difference in FVC and a −19-mL (95% CI −28 to −9 mL) difference in FEV1 in men.

**Conclusions:**

Inverse associations between glycemic measures and lung function were observed. Men seem more susceptible to the alteration in FVC and FEV1 than women.

## Abstract (ja)

**【背景】:**

糖代謝の状態と肺機能の負の関連は報告されているが、その用量依存関係については未だ十分調査がされていない。今回の研究では、これに連続的な負の関連があることを仮説とし日本人健診集団においてそれを検証した。

**【方法】:**

この横断研究は2008年から2011年に東京都済生会中央病院にて健診を受診した3,161人の日本人成人を対象として行われた。糖代謝状態の指標としてヘモグロビンA1c (HbA1c)と空腹時血糖値(FPG)を、肺機能検検査として努力性肺活量(FVC)と1秒量(FEV1)を用い、これらの関連を調べるため多変量線形回帰分析を行った。

**【結果】:**

男女ともに糖代謝の状態と肺機能は負の関連を認めたが、多変量調整後、女性では関連は減弱した。HbA1cの1％の上昇は、女性ではFVCの-52ml (95％信頼区間：-111 to 8) の変化とFEV1の-25ml (95％信頼区間：-75 to 25) の変化と関連し、男性ではFVCの-128ml (95％信頼区間：-163 to -94) の変化とFEV1の-73ml (95％信頼区間：-101 to -44)　の変化と関連した。FPGの10mg/dlの上昇は、女性ではFVCの-11ml (95％信頼区間：-29 to 8) の変化とFEV1の-8ml (95％信頼区間：-24 to 7) の変化と関連し、男性ではFVCの-32ml (95％信頼区間：-44 to -21) の変化とFEV1の-19ml (95％信頼区間：-28 to -9) の変化と関連した。

**【結論:**

】糖代謝の状態と肺機能の連続的な負の関連が観察された。糖代謝による肺機能の変化は男性の方がより大きいと考えられた。

## INTRODUCTION

Diabetes mellitus is a chronic disease for which the burden is increasing in many countries. A dramatic increase in the number of diabetic people has been observed in the past several decades. The worldwide prevalence of diabetes in 2010 was estimated to be 6.4%, affecting 285 million adults.^1^ It is projected that the number will increase to 439 million by 2030.^1^

The associations of diabetes with diseases in various organs and organ systems, such as the eyes, nerves, kidneys, and cardiovascular system, have been established. The lungs have been suggested as possible target organs that could be affected by diabetes.^2^^,^^3^ Past studies have reported that diabetes is associated with impaired lung function,^4^^–^^13^ and the dose-response relationship between glycemic measures and impaired lung function has been reported recently.^6^^,^^10^^–^^12^ Some studies categorized study participants into quartiles^6^ or quintiles^11^ of glycemic measures to examine the relationship between glycemic measures and lung function. Others^10^^,^^12^ used categories based on conventional cutoff values, such as fasting plasma glucose (FPG) of <100 mg/dL (normal), 100–109 mg/dL (high-normal), and 110–125 mg/dL (impaired fasting glucose)^12^; or non-diabetes, diabetes with HbA1c of <8.0%, and diabetes with HbA1c of ≥8.0%.^10^ However, few studies have examined the relationship between glycemic measures and lung function tests in a continuous manner. Given that the association of glycemic status with impaired lung function is well-known, grasping the effect size of the dose-response relationship might be a subject for further study.

In the present study, we hypothesized that there is a continuous and inverse association between glycemic status and lung function and examined the hypothesis in a large sample from a health screening program, which included both diabetic and non-diabetic individuals.

## METHODS

### Study participants

This was a cross-sectional study performed at the medical checkup unit of Saiseikai Central Hospital in Tokyo, Japan. The study population was composed of participants (*n* = 3276) who underwent a health screening program focusing on metabolic syndrome from May 2008 to December 2011. They were generally residents of neighboring areas and visited the hospital not for symptomatic diseases but for a health check. Of the 3276 participants, 11 participants were excluded because of missing data. We also excluded the top and bottom 0.5% of the results of glycemic measures (hemoglobin A1c [HbA1c] and FPG) and lung function tests (forced vital capacity [FVC] and forced expiratory volume in 1 second [FEV1]) (*n* = 104). The reason for the exclusion was to avoid the influence of extreme or unreliable values on the analysis. The study protocol was reviewed and approved by the ethics committee of Saiseikai Central Hospital.

### Glycemic measures

Blood samples were obtained 12 hours or more after the last caloric intake. Plasma glucose levels were measured using GA08 (A&T Co., Kanagawa, Japan). HbA1c levels were measured using an HPLC analyser (HLC-723G8; Tosoh, Tokyo, Japan). The HbA1c data were converted to the equivalent values of the National Glycohemoglobin Standardization Program according to the statement of the Japan Diabetes Society.^14^

### Definitions of diabetes

Diabetes was defined as any of the following: self-reported diabetes or use of anti-diabetic medications based on the questionnaire, fasting plasma glucose of ≥126 mg/dL, or HbA1c of ≥6.5%.

### Lung function tests

Lung function tests, including measurement of FVC and FEV1, were performed using a spirometer (Spiro Sift SP-350 COPD; Fukudadenshi, Tokyo, Japan) by trained personnel without knowledge of the purpose of the present study.

### Other variables

Variables which could be potential confounders were identified from past studies.^4^^–^^10^^,^^13^^,^^15^ Age, sex, weight, height, body mass index (BMI), blood pressure, education, occupation, smoking status, physical activity, and white blood cell (WBC) count were candidate variables. Available information on these variables was gathered from the health screening data. Blood samples and anthropometric measurements were obtained under a fasting condition. Blood pressure was measured in a sitting posture using an automated sphygmomanometer (Udex-Twin; ELK Corp, Osaka, Japan). Information on smoking status was obtained by a self-administered questionnaire. The items in the questionnaire included current smoking status (yes/no), lifetime smoking duration (none, <10, 10–19, 20–29, 30–39, or ≥40 years), and daily smoking quantity (none, 1–5, 6–10, 11–20, 21–30, or ≥31 cigarettes per day). Although the classification used in the questionnaire was detailed, we simply categorized the participants into three groups (non-smoker, past smoker, or current smoker) according to the information. If current smoking status was ‘yes’, a participant was categorized as a ‘current smoker’. If current smoking status was ‘no’ and either of lifetime smoking duration or daily smoking quantity was not ‘none’, a participant was categorized as a ‘past smoker’. If current smoking status was ‘no’ and both lifetime smoking duration and daily smoking quantity were ‘none’, a participant was categorized as a ‘non-smoker’.

### Statistical analysis

All analyses were stratified by sex because lung volume was substantially different between men and women. Age-adjusted Pearson correlations of lung function tests (FVC and FEV1) with continuous variables were calculated. To evaluate whether lung function tests are associated with glycemic measures, we performed multivariable-adjusted linear regressions with glycemic measures (HbA1c and FPG) as dependent variables and lung function tests (FVC and FEV1) as independent variables. In the multivariable models, confounders were included if they were significant at a 0.05 level in univariate analyses. As a result, the models were adjusted for age, height, weight, smoking status, and log-transformed WBC count. The WBC count was log-transformed because of its skewed distribution. Body mass index was not included in the models because it did not have significant associations with lung function. A squared term of the height, which had been included in the models used in past studies,^7^^,^^8^ was not included in the present study, since adding the term did not improve model fit in the analyses of FVC or FEV1. The sex difference in the association of glycemic measures with lung function tests was statistically tested by adding an interaction term (HbA1c*sex or FPG*sex) into the regression models.

Statistical analyses were performed using STATA software version 11 (StataCorp, College Station, TX, USA). All statistical tests were two-sided, and *P*-values less than 0.05 were considered statistically significant.

## RESULTS

Patients’ characteristics are shown in Table 1. A total of 986 women and 2175 men were included. The average age was 55.2 years in women and 56.5 years in men. The respective average FPG and HbA1c levels were 97 mg/dL and 5.6% in women and 107 mg/dL and 5.8% in men. Regarding the lung function tests, the respective average FVC and FEV1 were 2759 and 2290 mL in women and 3846 and 3100 mL in men.

**Table 1.** Characteristics of study participants

Characteristics	Women (*n* = 986)	Men (*n* = 2175)
Age, years	55.2 (12.1)	56.5 (11.9)
BMI, kg/m^2^	21.4 (3.2)	23.9 (2.9)
Height, cm	157.1 (5.6)	169.7 (5.9)
Weight, kg	52.8 (8.1)	69.0 (9.8)
Systolic blood pressure, mm Hg	116 (19)	123 (17)
Diastolic blood pressure, mm Hg	73 (12)	78 (11)
Antihypertensive treatment, *n* (%)	128 (13.0)	468 (21.5)
FPG, mg/dL	97 (14)	107 (20)
HbA1c, %	5.6 (0.5)	5.8 (0.7)
Diabetes, *n* (%)	59 (6.0)	371 (17.1)
Glucose-lowering treatment, *n* (%)	31 (3.1)	193 (8.9)
WBC, count/µL	5000 (4200–5900)	5600 (4800–6600)
Smoking status		
Non-smoker	833 (84.5)	1160 (53.3)
Past smoker	63 (6.4)	489 (22.5)
Current smoker	90 (9.1)	526 (24.2)
FVC, mL	2759 (515)	3846 (701)
FEV1, mL	2290 (453)	3100 (624)

BMI, body mass index; FEV1, forced expiratory volume in 1 second; FPG, fasting plasma glucose; FVC, forced vital capacity; HbA1c, hemoglobin A1c; WBC, white blood cell.

Data are presented as mean (standard deviation) or median (interquartile range) for continuous variables and count (%) for categorical variables.

Pearson correlation coefficients of FVC and FEV1 with patients’ characteristics are shown in Table 2. FVC was negatively correlated with age in both women and men. After adjustment for age, FVC was positively correlated with height and weight and negatively correlated with WBC count in both sexes. In women, FVC was not significantly correlated with HbA1c (*r* = −0.06) or FPG (*r* = −0.02), whereas in men FVC was negatively and significantly correlated with HbA1c (*r* = −0.16) and FPG (*r* = −0.11). Regarding FEV1, the results were similar to those of FVC. FEV1 was correlated with age, height, weight, and WBC count in both sexes. Significant correlations with HbA1c and FPG were observed only in men (HbA1c: women, *r* = −0.03; men, *r* = −0.12, FPG: women, *r* = −0.02; men, *r* = −0.08).

**Table 2.** Age-adjusted, sex-specific Pearson correlation coefficients of FVC and FEV1 with patients’ characteristics

Characteristics	FVC				FEV1			
	
	Women (*n* = 986)	*P*-value	Men (*n* = 2175)	*P*-value	Women (*n* = 986)	*P*-value	Men (*n* = 2175)	*P*-value
Age^a^	−0.54	<0.001	−0.54	<0.001	−0.61	<0.001	−0.63	<0.001
BMI	−0.02	0.539	−0.03	0.176	0.02	0.546	0.00	0.979
Height	0.43	<0.001	0.40	<0.001	0.38	<0.001	0.35	<0.001
Weight	0.16	<0.001	0.17	<0.001	0.18	<0.001	0.17	<0.001
Systolic blood pressure	−0.03	0.413	−0.04	0.043	−0.01	0.661	−0.02	0.367
Diastolic blood pressure	0.03	0.309	−0.01	0.661	0.05	0.153	0.01	0.651
FPG	−0.02	0.435	−0.11	<0.001	−0.02	0.528	−0.08	<0.001
HbA1c	−0.06	0.070	−0.16	<0.001	−0.03	0.349	−0.12	<0.001
Log WBC count	−0.07	0.024	−0.12	<0.001	−0.12	<0.001	−0.16	<0.001

BMI, body mass index; FEV1, forced expiratory volume in 1 second; FPG, fasting plasma glucose; FVC, forced vital capacity; HbA1c, hemoglobin A1c; WBC, white blood cell.

^a^Unadjusted correlation.

The associations of glycemic measures with FVC and FEV1 are given in Figure 1a and Figure 1b. Inverse associations were graphically observed in both sexes. In the linear regression analyses (Table 3), a 1% absolute increase in HbA1c was associated with a −297-mL (95% confidence interval [CI] −364 to −231 mL) difference in FVC in women and a −287-mL (95% CI −328 to −246 mL) difference in men. A 10-mg/dL increase in FPG was associated with a −67-mL (95% CI −89 to −46 mL) difference in FVC in women and a −73-mL (95% CI −87 to −59 mL) difference in men. However, in the multivariable-adjusted models, the values were attenuated in both sexes and became non-significant in women. A 1% absolute increase in HbA1c was associated with a −52-mL (95% CI −111 to 8 mL) difference in FVC in women and a −128-mL (95% CI −163 to −94 mL) difference in men. A 10-mg/dL increase in FPG was associated with a −11-mL (95% CI −29 to 8 mL) difference in FVC in women and a −32-mL (95% CI −44 to −21 mL) difference in men. When diabetes was analyzed as a categorical variable, the multivariable-adjusted model showed that the presence of diabetes was associated with a non-significant −74-mL (95% CI −182 to 33 mL) difference in FVC in women and a significant −208-mL (95% CI −270 to −147 mL) difference in men.

**Figure 1a.**
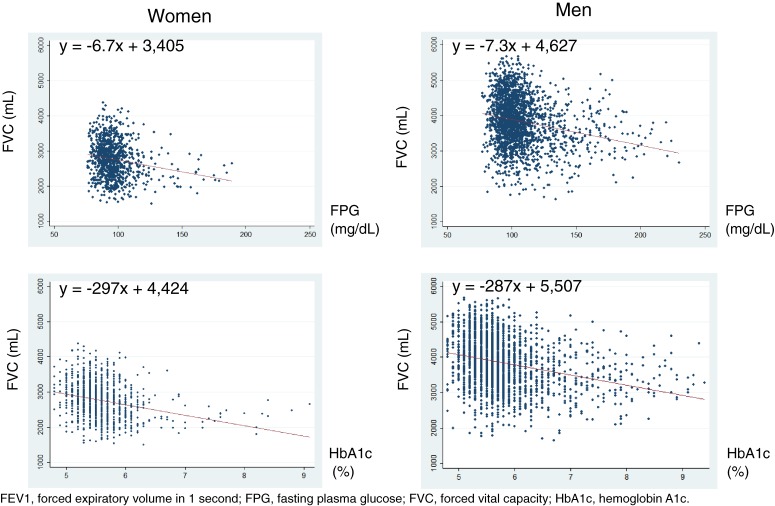
Regression analyses of FVC in relation to glycemic measures.

**Figure 1b.**
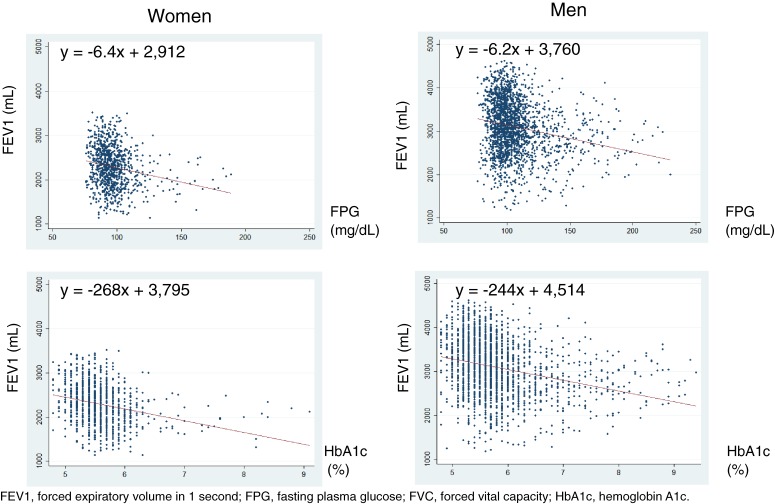
Regression analyses of FEV1 in relation to glycemic measures.

**Table 3.** Sex-specific, multivariable adjusted associations of FVC and FEV1 with glycemic measures

Traits	FVC					FEV1				
	
	Women		Men		*P* for interaction by sex	Women		Men		*P* for interaction by sex
			
	Beta (mL)	(95% CI)	Beta (mL)	(95% CI)		Beta (mL)	(95% CI)	Beta (mL)	(95% CI)	
Continuous traits										
HbA1c										
(1% absolute increase)										
Base model	−297	(−364 to −231)	−287	(−328 to −246)	0.831	−268	(−327 to −210)	−244	(−281 to −208)	0.562
Age-adjusted	−59	(−123 to 5)	−141	(−178 to −104)	<0.001	−25	(−78 to 28)	−88	(−118 to −57)	<0.001
Fully adjusted^a^	−52	(−111 to 8)	−128	(−163 to −94)	<0.001	−25	(−75 to 25)	−73	(−101 to −44)	<0.001

FPG										
(increase of 10 mg/dL)										
Base model	−67	(−89 to −46)	−73	(−87 to −59)	0.675	−64	(−83 to −45)	−62	(−74 to −49)	0.866
Age-adjusted	−8	(−28 to 12)	−34	(−46 to −21)	0.001	−5	(−22 to 11)	−20	(−30 to −10)	0.001
Fully adjusted^a^	−11	(−29 to 8)	−32	(−44 to −21)	<0.001	−8	(−24 to 7)	−19	(−28 to −9)	0.001

Categorical traits										
Diabetes										
Base model	−310	(−445 to −176)	−487	(−563 to −411)	0.054	−260	(−378 to −141)	−401	(−469 to −333)	0.086
Age-adjusted	−89	(−205 to 28)	−253	(−321 to −186)	0.004	−37	(−134 to 59)	−151	(−207 to −96)	0.003
Fully adjusted^a^	−74	(−182 to 33)	−208	(−270 to −147)	0.001	−30	(−120 to 59)	−110	(−162 to −59)	0.002

CI, confidence interval; FEV1, forced expiratory volume in 1 second; FPG, fasting plasma glucose; FVC, forced vital capacity; HbA1c, hemoglobin A1c.

^a^Adjusted for age, height, weight, smoking status, and log WBC count.

Regarding FEV1, the findings were similar, although the calculated regression coefficients were smaller than those in the analyses of FVC. In the multivariable-adjusted models, a 1% absolute increase in HbA1c was associated with a −25-mL (95% CI −75 to 25 mL) difference in FEV1 in women and a −73-mL (95% CI −101 to −44 mL) difference in men. A 10-mg/dL increase in FPG was associated with a −8-mL (95% CI −24 to 7 mL) difference in FEV1 in women and a −19-mL (95% CI −28 to −9 mL) difference in men. When diabetes was analyzed as a categorical variable, the multivariable-adjusted model showed that the presence of diabetes was associated with a non-significant −30-mL (95% CI −120 to 59 mL) difference in FEV1 in women and a significant −110-mL (95% CI −162 to −59 mL) difference in men.

Significant sex differences in the associations between glycemic measures and lung function tests were observed in the multivariable-adjusted models (Table 3).

## DISCUSSION

The present cross-sectional study analyzed the associations of glycemic status with lung function in Japanese adults who participated in a health screening program. We observed that FPG and HbA1c were negatively and significantly correlated with FVC and FEV1 in men. In women, weak and negative correlations were observed, but they were not significant. In the scatterplots with a fitted regression line (Figure 1a and Figure 1b), inverse associations of FVC and FEV1 with HbA1c and FPG were observed. However, after adjustment for confounders, the associations remained significant only in men, while they became attenuated and non-significant in women.

Past cross-sectional studies^4^^–^^12^ have investigated the association of diabetes with impaired lung function and reported that individuals with diabetes have lower FVC and FEV1 than those without. Although the dose-response relationships of glycemic measures with lung function tests have been reported in several studies,^6^^,^^10^^–^^12^ evidence is still insufficient. The Toranomon Hospital Health Management Center Study 9,^12^ which categorized Japanese men without diabetes into three groups according to their HbA1c levels (<5.7%, 5.7–5.9%, 6.0–6.4%), reported that higher HbA1c levels were associated with lower FVC and FEV1, while the associations of FPG with lung function tests were not significant. This study did not include women or diabetic participants. As for studies that included both non-diabetic and diabetic individuals, the Framingham Offspring Cohort Study,^6^ which categorized participants into quartiles according to their FPG levels, showed that higher FPG was associated with lower FVC and FEV1. Another study analyzing the Third National Health and Nutrition Examination Study,^11^ which categorized participants into quintiles according to FPG and HbA1c levels, showed that higher FPG and HbA1c levels were associated with lower FVC and FEV1. However, in these studies,^6^^,^^11^^,^^12^ participants with diabetes were included in the highest group of the categorization. The dose-response relationship in participants with diabetes has not been assessed. In the present study, we observed linear and inverse associations between glycemic measures and lung function tests in a wide range of HbA1c and FPG, which included both diabetic and non-diabetic individuals.

Regarding the magnitude of the impairment of lung function, a systematic review reported that past cross-sectional studies have mostly found that diabetes was associated with 3%–10% lower values in lung volumes of FVC and FEV1.^16^ In our estimation, individuals with diabetes had 2%–5% lower lung volumes than those without. Our findings seem consistent with the past findings and further revealed the magnitude of the impairment according to glycemic measures. As for sex differences, we observed significant associations of glycemic measures with lung function tests in men but not in women in the multivariable-adjusted models. Findings on tests for interaction by sex were statistically significant. Although the non-significant results in women could be explained by the small sample size, it is likely that the associations were nevertheless stronger in men than in women. The mechanisms underlying the sex difference remain unclear. Sex hormones, behavioral factors, or differences in occupational exposures may also be involved in the effect modification by sex. However, considering that sex differences have been observed in associations between diabetes and the development of its complications,^17^^,^^18^ a sex difference may indeed exist in the association between glycemic status and impaired lung function.

Several explanations for the association between hyperglycemia and impaired lung function have been proposed. One explanation is that hyperglycemia induces microangiopathy in the lungs. A past study^10^ reported that lung function measurements were correlated with diabetic microangiopathy in the eyes, nerves, and kidneys. However, longitudinal studies have not proven a definitive causal association,^5^^,^^7^^,^^9^^,^^13^ and the opposite direction of the association has also been proposed.^16^ Several longitudinal studies have reported that impaired lung function preceded the incidence of diabetes.^15^^,^^19^^–^^21^ Hypoxia-induced insulin resistance^22^^,^^23^ or low physical activity^24^ caused by pulmonary problems are possible explanations. It has also been suggested that systemic inflammation caused by diabetes might be associated with impaired lung function.^13^ The present study found that WBC count increased with the glycemic measures, which might reflect systemic inflammation. However, when the WBC count was added into the multivariable models, the associations were not attenuated.

Although it remains unclear whether or not the association between glycemic status and impaired lung function is causal, the association has an important clinical implication. The magnitude of the difference in lung volumes attributable to poor glycemic status might be minimal and negligible for individuals; however, as the number of diabetic people increases, a large number of diabetic people exposed to a small risk may generate a non-negligible number of instances of lung disease.^25^ Coexisting conditions with diabetes, such as aging or smoking, may accelerate impairment of lung function, which may eventually lead to acute or chronic lung disease.

Several limitations in the present study warrant mention. First, we were not able to evaluate the long-term effect of exposure to hyperglycemia on the lungs because data on duration of diabetes or long-term changes in glycemic status were not available. Second, people who underwent the health screening might be more health-conscious than those who did not. Therefore, the characteristics of the study participants in the present study might be different from a general population, which limits the generalizability of the study. In addition, there may have been unidentified confounders in the association between glycemic status and impaired lung function. As mentioned above, the results were not adjusted for education, occupation, or physical activity levels, since these data were not available in the present study. Exposures to environmental or occupational contaminants, which were not measured in the present study, might also affect the association. Despite these limitations, the present study reported the association with a large sample size including both diabetic and non-diabetic individuals. In addition, the analyses were stratified by sex, which enabled us to contrast the sex differences in the associations.

In summary, linear and inverse associations between glycemic measures and lung function tests were observed in the present study. Men seem more susceptible than women to alterations in FVC and FEV1. Although the magnitude of the difference in lung function might be subclinical, we should keep in mind the possibility of impaired lung function in daily diabetes practice.

## ONLINE ONLY MATERIAL

Abstract in Japanese.
