# Supplementary material for: Association of Glycemic Status With Impaired Lung Function Among Recipients of a Health Screening Program: A Cross-Sectional Study in Japanese Adults
Source: J Epidemiol. 2014 Sep 5;24(5):410–6. doi: 10.2188/jea.JE20140016 (PMC4150013; doi:10.2188/jea.JE20140016)
Supplement: Abstract in Japanese. [file je-24-410-s001.pdf]

糖代謝の状態と肺機能の関連：日本人健診受診者における横断研究

壁谷悠介、加藤清恵、富田益臣、香月健志、及川洋一、島田朗
東京都済生会中央病院

【背景】糖代謝の状態と肺機能の負の関連は報告されているが、その用量依存関係については未だ十分調査がされていない。今回の研究では、これに連続的な負の関連があることを仮説とし日本人健診集団においてそれを検証した。
【方法】この横断研究は 2008 年から 2011 年に東京都済生会中央病院にて健診を受診した 3,161 人の日本人成人を対象として行われた。糖代謝状態の指標としてヘモグロビン A1c (HbA1c)と空腹時血糖値(FPG)を、肺機能検検査として努力性肺活量(FVC)と 1 秒量(FEV1)を用い、これらの関連を調べるため多変量線形回帰分析を行った。
【結果】男女ともに糖代謝の状態と肺機能は負の関連を認めたが、多変量調整後、女性では関連は減弱した。HbA1c の 1%の上昇は、女性では FVC の-52ml (95%信頼区間：-111 to 8) の変化と FEV1 の-25ml (95%信頼区間：-75 to 25) の変化と関連し、男性では FVC の-128ml (95%信頼区間：-163 to -94) の変化と FEV1 の-73ml (95%信頼区間：-101 to -44) の変化と関連した。FPG の 10mg/dl の上昇は、女性では FVC の-11ml (95%信頼区間：-29 to 8) の変化と FEV1 の-8ml (95%信頼区間：-24 to 7) の変化と関連し、男性では FVC の-32ml (95%信頼区間：-44 to -21) の変化と FEV1 の-19ml (95%信頼区間：-28 to -9) の変化と関連した。
【結論】糖代謝の状態と肺機能の連続的な負の関連が観察された。糖代謝による肺機能の変化は男性の方がより大きいと考えられた。

キーワード：糖尿病、糖代謝状態、肺機能
